# Driver genes exome sequencing reveals distinct variants in African Americans with colorectal neoplasia

**DOI:** 10.18632/oncotarget.26721

**Published:** 2019-04-05

**Authors:** Hassan Ashktorab, Hamed Azimi, Sudhir Varma, Edward L. Lee, Adeyinka O. Laiyemo, Michael L. Nickerson, Hassan Brim

**Affiliations:** ^1^ Department of Medicine, Cancer Center, Howard University, Washington, DC, USA; ^2^ Department of Pathology, Howard University College of Medicine, Washington, DC, USA; ^3^ Hithru Analytics, LLC, Silver Spring, MD, USA; ^4^ Laboratory of Translational Genomics, National Cancer Institute, Bethesda, MD, USA

**Keywords:** colon, targeted sequencing, African Americans, actionable, druggable

## Abstract

**Background:**

Colorectal cancer (CRC) is the third leading cause of cancer-related deaths in the United States. African Americans are disproportionately affected by CRC. Our hypothesis is that driver genes with known and novel mutations have an impact on CRC outcome in this population. Therefore, we investigated the variants’ profiles in a panel of 15 CRC genes.

**Patients & Methods:**

Colorectal specimens (n=140) were analyzed by targeted exome sequencing using an Ion Torrent platform. Detected variants were validated in 36 samples by Illumina sequencing. The novel status of the validated variants was determined by comparison to publicly available databases. Annotated using ANNOVAR and *in-silico* functional analysis of these variants were performed to determine likely pathogenic variants.

**Results:**

Overall, 121 known and novel variants were validated: *APC* (27%), *AMER1* (3%)*, ARID1* (7%), *MSH3* (12%), *MSH6* (10%), *BRAF* (4%), *KRAS* (6%), *FBXW7* (4%), *PIK3CA* (6%), *SMAD4* (5%), *SOX9* (2%), *TCF7L2* (2%), *TGFBR2* (5%), *TP53* (7%). From these validated variants, 12% were novel in 8 genes *(AMER1, APC, ARID1A, BRAF, MSH6, PIK3CA, SMAD4,* and *TCF7L2*). Of the validated variants, 23% were non-synonymous, 14% were stopgains, 24% were synonymous and 39% were intronic variants.

**Conclusion:**

We here report the specifics of variants’ profiles of African Americans with colorectal lesions. Validated variants showed that Tumor Suppressor Genes (TSGs) *APC* and *ARID1* and DNA Mismatch repair (MMR) genes *MSH3* and *MSH6* are the genes with the highest numbers of validated variants. Oncogenes *KRAS and PIK3CA* are also altered and likely participate in the increased proliferative potential of the mutated colonic epithelial cells in this population.

## INTRODUCTION

Colorectal Cancer (CRC) is the third leading cause of cancer-related deaths in the US. Its incidence and mortality are higher in African Americans (AAs) than other ethnic groups in the US. The reasons for this disparity are not yet well established [1]. It is thought that the underlying factors behind this disparity are multiple (diet, lifestyle, microbiome, socioeconomic, healthcare access and genetic predispositions). Cancer driver mutations play an important role in the carcinogenic process [2, 3]. There are limited next generation sequencing studies in African Americans with cancer in general and with CRC in particular [4–6]. However, none of these studies investigated cancer specimens along with pre-neoplastic lesions.

Molecularly, CRCs are categorized into those with microsatellite instability (MSI) which are located primarily in the right colon and frequently associate with the CpG island methylator phenotype (CIMP) and those that are microsatellite stable (MSS) but are chromosomally unstable [7–9]. MSI characterizes 10–15% of sporadic CRCs and has been related to a better prognosis compared with MSS colorectal cancer patients [7, 10]. Most MSI CRCs have been assigned previously to defects in *MLH1* and *MSH2* genes within the DNA Mismatch Repair (MMR) genes machinery. However recent attention has been given to other genes such as *MSH3* and *MSH6* that were found to be altered and associated with a different category of microsatellite instability within the genome, primarily at tetranucleotide repeats [11].

Several pathogenic gene panels that are frequently mutated in CRC have been designed for targeted sequencing. We examined 15 genes associated with CRC using a Personal Genome Machine (PGM; Ion Torrent-ThermoFisher Scientific; Waltham, MA) sequencing platform for variant discovery, and a MiSeq (Illumina; San Diego, CA) sequencing platform for validation. The 15 genes correspond to two DNA MMR genes, 6 oncogenes and 7 tumor suppressor genes (TSGs). The DNA MMR gene *MSH3* have acquired more attention recently in CRC patients as variants in this gene were found to be prevalent in African Americans (50 to 60%) and associate with poor prognosis [11, 12] in contrast with MSI-H phenotype that is driven by altered *MLH1* and *MSH2* alterations. The 15 genes panel also consisted of 6 oncogenes namely: *BRAF, NRAS, KRAS, PIK3CA*, *SMAD4* and *SOX9*. *KRAS* is involved in the pathogenesis of many different malignant tumors, including lung, pancreatic, and colon cancers [13]. Around 30 to 40% of CRCs have *KRAS* variants [14]. *NRAS* is a member of a family of oncoproteins that are commonly mutated in cancer. Activating variants in *NRAS* occur in a subset of CRC but little is known about how the mutant protein contributes to the onset and progression of the disease [14]. *BRAF* is mutated in 4 to 12% of unselected CRC, particularly those with high microsatellite instability [15]. *BRAF* mutations in CRC are associated with distinct clinical characteristics and a worse prognosis [16]. *PIK3CA* encodes for the catalytic p110-alpha subunit of Phosphatidylinositol 3-Kinase (PI3K) alpha, which orchestrates cell responses including cell proliferation, survival, migration and morphology [17]. Activating mutations in *PIK3CA* were reported in 10 to 15% of colorectal carcinomas [18]. *SOX9* has been widely studied in the context of development and cell lineage determination in various tissues. Recent studies have indicated tissue- and context-specific roles of this gene [19].

The gene panel contained 7 tumor suppressor genes (*AMER1, APC, ARID1, FBXW7, TCF7L2, TGFBR2,* and *TP53)*. *APC* is one of the key genes in the initiation of polyp formation [20] in both familial adenomatous polyposis (FAP) and FAP-like sporadic CRCs [21]. Current studies have shown mutations of *APC* in many cancers including CRC. Several studies have suggested that chromosome 18q loss is a critical event during CRC progression and that the *SMAD4* tumor suppressor is the primary target for inactivation [22]. Clinical studies have shown that patients retaining heterozygosity at the 18q locus benefit significantly better from treatment with 5-Fluorouracil than patients with loss of heterozygosity (LOH) at this site [23]. *AT-Rich Interactive Domain 1A* (*ARID1A*) has recently been identified as a novel tumor suppressor in various tumor types. Loss of *ARID1A* expression is uncommon and not associated with oncologic outcome but may be related to less invasive pathologic features in CRC [24]. Most CRCs with microsatellite instability (MSI-H) have mutations in a microsatellite sequence encoding *Transforming Growth Factor β Receptor II* (*TGFBR2*). Therefore, it is understood that TGFBR2 is defective in these tumors, even though CRC cells with *TGFBR2* variants have been described to remain sensitive to TGFβ [25]. Resistance to growth inhibition by TGFβ is standard in a variety of human cancers, emphasizing the importance of intracellular pathways mediated by this polypeptide to the neoplastic process [26]. *FBXW7* variants occur in a variety of human cancers including CRC [27]. *FBXW7* and *TP53* are tumor suppressors intensively implicated in colorectal carcinogenesis [28]. *FBXW7* constitutes one of the four subunits of SCF (SKP1-cullin-F-box)-E3 ubiquitin-protein ligase complex, which functions in phosphorylation-dependent ubiquitination [27]. About half of all CRCs show *TP53* gene variants, with lower frequencies in proximal tumors and higher frequencies in distal colon and rectal tumors and in those with the microsatellite instability or methylator phenotypes [28]. *AMER1* regulates the distribution of the tumor suppressor APC between microtubules and the plasma membrane [29]. It is frequently mutated in colorectal cancer tumors [30]. TCF7L2 is a transcription factor of which polymorphisms have been associated with cancers including colon and prostate [31]. However, this gene’s polymorphisms have been intensively studied in the context of diabetes-associated disorders [32].

In the present study, we examined and validated variants in a panel of cancer genes and assessed their association with disease characteristics in African Americans with colorectal neoplastic lesions.

## RESULTS

### Clinical and pathological characteristics of patients

**Figure 1:**
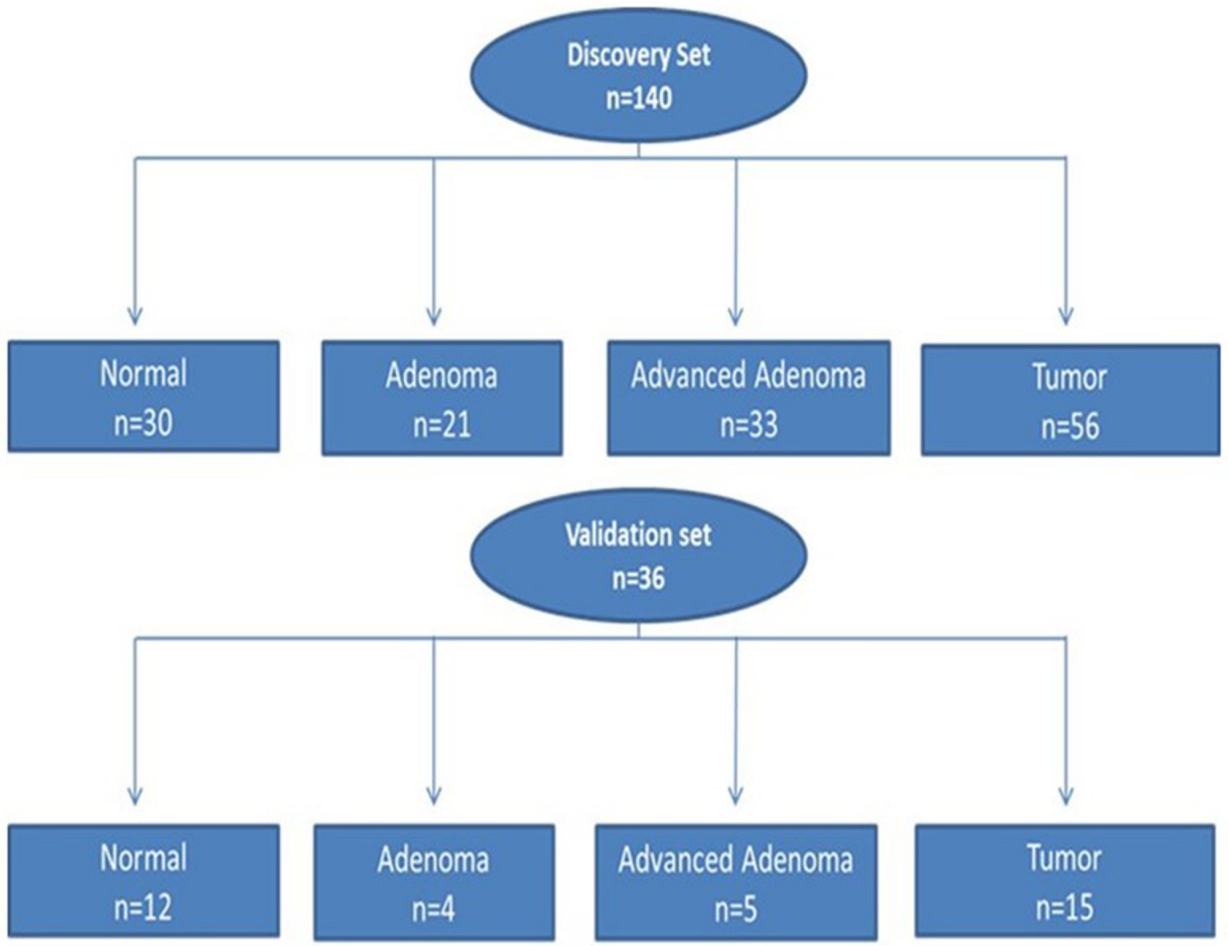
Flow chart of patient selection for both discovery and validation sets for somatic variants analysis Discovery set: 140 samples (n = 123 patients) and validation set: 36 samples (n = 26 patients).

A: Discovery set: The characteristics of the 123 patients from whom the 140 samples were collected are reported in Figure 1. The patients consisted of 69 (57%) males. The age range at the time of diagnosis was 24 to 95 years, with a median of 61 years. With regard to cancer stage, 11% (6/56) were stage I, 34% (19/56) were stage II, 29% (16/56) were stage III, and 4% (2/56) were stage IV (23% (13/56) had no staging data).
B: Validation set: Contained a subset of the discovery set and was made of 36 samples from 26 patients. There were 10 (38%) females and 16 (62%) males. With regard to cancer stage, 35% (9/26) were stage II, 15% (4/26) were stage III and 8% (2/26) stage IV, and 42% (11/26) had no staging data. The age range was from 41 to 88 with median age of 61 years (Table 1).

**Figure 2:**
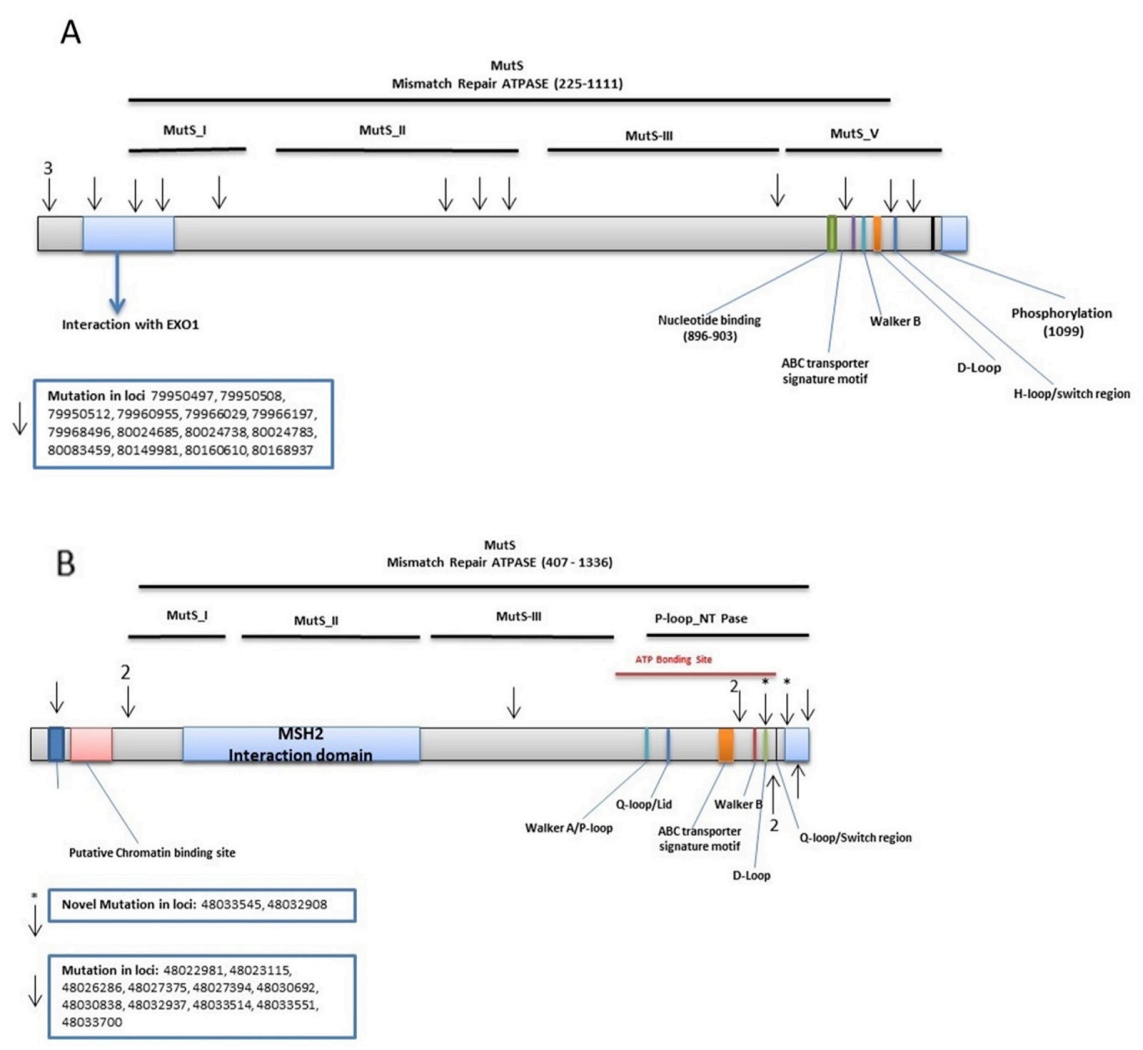
Distribution of validated variants per targeted MMR genes **(A)** *MSH3* **(B)** *MSH6*.

**Table 1:** Clinico-pathological characteristics of the validation set (26 Patients & 36 Samples)

Sample ID	Age	Sex	Type of Tissue	Location	TNM	STAGING
**CC1018N**	84	F	N	RIGHT	N	NA
**CC1018**	84	F	T	LEFT	T3N1b	II
**CC1024**	73	F	T	RIGHT	T3N1MX	III
**CC1028**	42	M	T	LEFT	T3N0MX	II
**CC1028N**	42	M	N	RIGHT	N	NA
**CC1029**	51	F	T	RIGHT	T2N0MX	II
**CC1029N**	51	F	N	LEFT	N	NA
**CC1036**	63	M	T	LEFT	T3N2M1	IV
**CC1036N**	63	M	N	RIGHT	N	NA
**CC1038**	54	M	T	LEFT	T3N0Mx	II
**CC1038N**	54	M	N	RIGHT	N	NA
**CC1053**	50	F	T	RIGHT	T3N0M0	II
**CC1053N**	50	F	N	LEFT	N	NA
**CC1054**	53	M	T	RIGHT	T3N0M0	II
**CC1054N**	53	M	N	LEFT	N	NA
**CC1055**	79	F	AA	RIGHT	AA	NA
**CC1056**	66	M	T	LEFT	T1N1MX	III
**CC1056N**	66	M	N	RIGHT	N	NA
**CC1057**	88	M	T	LEFT	T3N2M0	III
**CC1057N**	88	M	N	RIGHT	N	NA
**CC1059**	60	F	T	RIGHT	T3N1MX	II
**CC1060**	53	F	T	LEFT	T3N2M1	IV
**CC1060N**	53	F	N	RIGHT	N	NA
**CC1061**	63	M	N	LEFT	N	NA
**CC1065**	41	M	T	LEFT	T3N0M0	II
**CC1109**	62	F	A	RIGHT	A	NA
**CC1258**	52	M	T	RIGHT	T3N1bMx	III
**CC1386**	70	M	T	LEFT	T3N0Mx	II
**CC1621N**	49	M	N	RIGHT	N	NA
**CC1680**	75	F	AA	RIGHT	AA	NA
**CC1681**	75	M	AA	LEFT	AA	NA
**CC1682**	71	M	AA	RIGHT	AA	NA
**CC1683**	45	M	A	RIGHT	AA	NA
**CC1698**	54	M	A	RIGHT	A	NA
**CC1720**	70	F	A	RIGHT	A	NA
**CC1721**	54	M	A	Missing	A	NA

N = Normal, T = CRC, A = Adenoma, AA = Advanced Adenoma

### Tumor suppressor genes

#### *APC* variants

**Figure 3:**
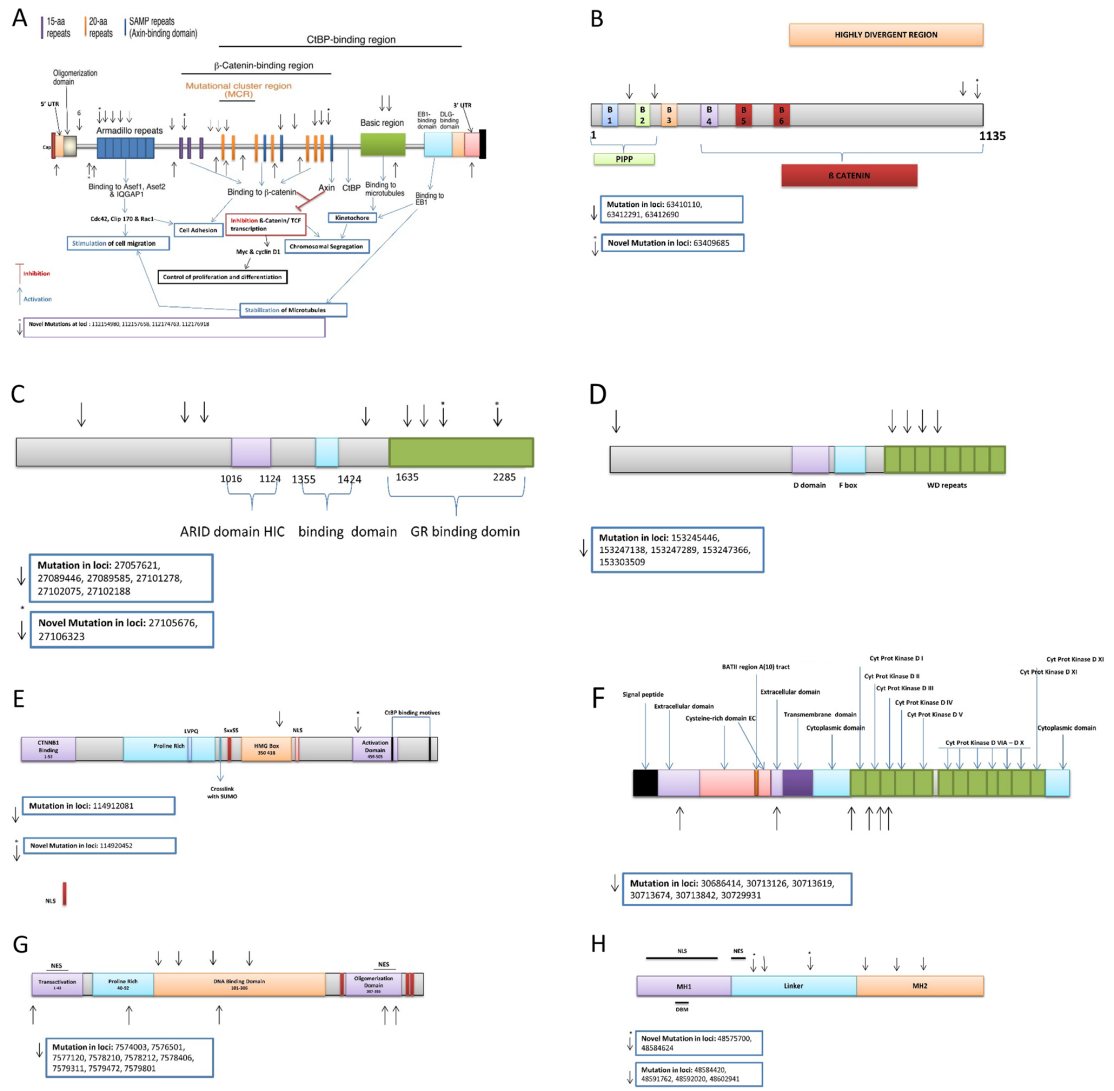
Tumor suppressor genes validated variants: (**A**) *APC,* (**B**) *AMER1,* (**C**) *ARID1,* (**D**) *FBXW7,* (**E**) *TCF7L2,* (**F**) *TGFBR2,* (**G**) *TP53,* (**H**) *SMAD4*.

We detected 944 variants in the discovery set, of which 822 are novel. The detailed information of these variants was recently published [5]. The Illumina sequencing led to the validation of 33 variants of which 4 were novel. Variant at locus chr5:112176918 with a G to A change had a frequency 0.03 (1/33, heterozygous) in advanced adenoma (Amino acid change from R to T). Variant at locus chr5: 112174763 with an A to T change had a frequency of 0.02 (1/56, heterozygous) in CRCs (Amino acid change from R to X). Variant at locus chr5:112154980 with a T to A change had a frequency 0.05 (1/21, heterozygous) in adenoma (Amino acid change from C to X). Variant at locus 112157658 with a G to T change had a frequency of 0.05 (1/21, heterozygous) in adenoma (Amino acid change from E to X). One variant was mapped in the 5’ UTR, 9 prior to ARM, 4 on the ARM, 3 prior to B-Catenin binding region, 12 in the B-Catenin binding region, 3 in the basic region, and 1 in the EB1 binding domain [5] (Table 2 and Figure 3A).

**Table 2:** Number of samples per validated variants in the targeted gene panel

Locus	Ref	Var	Gene	Variant type	Status (1=novel, 0=known)	AA-Normal Het (30)	AA-Normal Hom (30)	AA-Adenoma Het (21)	AA-Adenoma Hom (21)	AA-Ad. Adenoma Het (33)	AA-Ad. Adenoma Hom (33)	AA-CRC Het (56)	AA-CRC Hom (56)
63409685	C	T	AMER1	intronic	1	2	0	1	0	1	0	1	0
63410110	T	C	AMER1	synonymous SNV	0	8	16	10	9	9	13	8	33
63412291	C	G	AMER1	non-synonymous SNV	0	2	1	3	0	0	0	4	1
63412690	A	C	AMER1	non-synonymous SNV	0	13	6	9	2	13	2	27	10
112043384	T	G	APC	intronic	0	6	0	3	0	7	1	12	0
112103015	C	A	APC	stopgain	0	0	0	0	0	0	0	1	0
112116592	C	T	APC	stopgain	0	0	0	0	0	0	0	2	0
112128191	C	T	APC	stopgain	0	0	0	1	0	2	0	2	0
112136947	A	T	APC	intronic	0	3	0	2	0	3	0	3	0
112151261	C	T	APC	stopgain	0	0	0	0	0	1	0	1	0
112154942	C	T	APC	stopgain	0	0	0	1	0	1	0	1	0
112154980	T	A	APC	stopgain	1	0	0	1	0	0	0	0	0
112157658	G	T	APC	stopgain	1	0	0	1	0	0	0	0	0
112162854	T	C	APC	synonymous SNV	0	11	1	7	2	14	2	17	6
112164561	G	A	APC	synonymous SNV	0	16	5	14	6	20	7	27	16
112173553	T	G	APC	synonymous SNV	0	1	0	1	0	0	0	0	0
112173899	C	T	APC	non-synonymous SNV	0	1	0	1	0	2	0	3	0
112174096	C	A	APC	stopgain	0	0	0	0	0	1	0	0	0
112174763	A	T	APC	stopgain	1	0	0	0	0	0	0	1	0
112175023	A	G	APC	synonymous SNV	0	3	0	2	0	4	0	3	0
112175030	G	A	APC	non-synonymous SNV	0	1	0	0	0	0	0	2	0
112175069	C	T	APC	stopgain	0	0	0	1	0	0	0	2	0
112175207	G	T	APC	stopgain	0	0	0	0	0	0	0	2	0
112175399	A	T	APC	stopgain	0	0	0	0	0	2	0	1	0
112175576	C	T	APC	stopgain	0	0	0	0	0	3	0	0	0
112175639	C	T	APC	stopgain	0	0	0	2	0	4	0	2	0
112175770	G	A	APC	synonymous SNV	0	17	5	13	7	20	8	26	14
112176325	G	A	APC	synonymous SNV	0	19	4	14	6	21	6	26	17
112176541	C	G	APC	synonymous SNV	0	1	0	1	0	0	0	2	0
112176559	T	G	APC	synonymous SNV	0	18	4	13	7	19	8	26	17
112176756	T	A	APC	non-synonymous SNV	0	3	26	4	17	8	24	4	50
112176918	G	C	APC	non-synonymous SNV	1	0	0	0	0	1	0	0	0
112177171	G	A	APC	synonymous SNV	0	16	5	14	6	21	7	31	15
112178492	C	T	APC	synonymous SNV	0	0	0	0	0	1	0	1	0
112178795	G	A	APC	non-synonymous SNV	0	1	0	0	0	0	0	2	0
112178995	A	G	APC	synonymous SNV	0	7	0	3	0	6	0	6	2
112179909	C	A	APC	intronic	0	9	1	9	1	12	0	20	3
27057621	A	C	ARID1A	intronic	0	3	0	0	0	8	0	0	0
27089446	G	C	ARID1A	intronic	0	2	0	0	0	5	1	1	0
27089585	C	A	ARID1A	synonymous SNV	0	0	0	1	0	1	0	0	0
27101278	C	T	ARID1A	synonymous SNV	0	1	0	0	0	0	0	1	0
27102075	G	A	ARID1A	synonymous SNV	0	2	0	2	0	0	0	2	0
27102188	A	G	ARID1A	non-synonymous SNV	0	1	0	4	0	7	0	7	0
27105676	G	T	ARID1A	stopgain	1	0	0	0	0	0	0	1	0
27106323	C	T	ARID1A	synonymous SNV	1	1	0	0	0	0	0	4	0
140434597	G	A	BRAF	intronic	0	7	0	4	0	6	0	5	0
140449071	C	G	BRAF	intronic	0	7	15	9	7	20	8	28	18
140449150	T	C	BRAF	synonymous SNV	0	10	13	11	5	20	8	30	18
140481511	A	C	BRAF	intronic	0	2	0	2	0	0	0	3	0
140487360	G	A	BRAF	non-synonymous SNV	1	0	0	0	0	0	0	1	0
79950497	C	T	DHFR,MSH3	intronic	0	6	4	6	2	3	2	14	5
79950508	C	T	DHFR,MSH3	intronic	0	4	0	1	0	3	0	5	0
79950512	A	G	DHFR,MSH3	intronic	0	5	14	1	8	3	8	13	26
153245446	G	A	FBXW7	non-synonymous SNV	0	0	0	0	0	1	0	0	0
153247138	G	T	FBXW7	intronic	0	1	0	0	0	0	0	1	0
153247289	G	A	FBXW7	non-synonymous SNV	0	0	0	0	0	1	0	2	0
153247366	C	T	FBXW7	non-synonymous SNV	0	0	0	0	0	1	0	0	0
153303509	C	T	FBXW7	intronic	0	1	0	0	0	1	0	1	0
25362854	C	T	KRAS	intronic	0	11	3	12	0	10	0	24	5
25368462	C	T	KRAS	synonymous SNV	0	0	29	0	21	1	32	1	53
25398255	G	T	KRAS	non-synonymous SNV	0	0	0	0	0	1	0	0	0
25398281	C	T	KRAS	non-synonymous SNV	0	0	0	3	0	5	0	9	0
25398284	C	T	KRAS	non-synonymous SNV	0	0	0	3	0	2	0	12	0
25398285	C	A	KRAS	non-synonymous SNV	0	0	0	0	0	2	0	1	0
25398285	C	T	KRAS	non-synonymous SNV	0	0	0	0	0	3	0	2	0
79960955	G	A	MSH3	intronic	0	11	5	10	4	16	1	24	7
79966029	G	A	MSH3	synonymous SNV	0	6	1	11	0	5	2	16	0
79966197	G	A	MSH3	intronic	0	11	5	11	4	19	1	26	5
79968496	C	T	MSH3	intronic	0	5	3	3	3	11	1	13	3
80024685	C	A	MSH3	non-synonymous SNV	0	0	0	2	0	0	0	0	0
80024738	A	G	MSH3	non-synonymous SNV	0	0	0	0	0	2	0	0	0
80024783	G	A	MSH3	non-synonymous SNV	0	1	0	0	0	5	0	2	0
80083459	G	A	MSH3	synonymous SNV	0	1	0	0	0	2	0	1	0
80149981	A	G	MSH3	non-synonymous SNV	0	2	27	4	16	7	23	5	48
80160610	T	A	MSH3	intronic	0	5	2	4	0	4	1	12	1
80168937	G	A	MSH3	non-synonymous SNV	0	11	15	10	8	19	10	22	30
48022981	G	T	MSH6	intronic	0	10	3	12	1	11	1	23	4
48023115	T	C	MSH6	synonymous SNV	0	9	1	5	1	7	1	14	1
48026286	C	T	MSH6	synonymous SNV	0	4	0	2	0	4	0	7	0
48027375	T	C	MSH6	synonymous SNV	0	4	0	2	0	7	0	5	1
48030692	T	A	MSH6	synonymous SNV	0	0	0	0	0	1	0	0	0
48030838	A	T	MSH6	intronic	0	10	2	12	0	8	1	17	2
48032908	A	G	MSH6	intronic	1	1	0	1	0	0	0	2	0
48032937	T	C	MSH6	intronic	0	4	25	4	17	9	21	12	40
48033514	T	C	MSH6	intronic	0	0	0	2	0	2	0	1	0
48033545	A	C	MSH6	intronic	1	0	0	0	0	0	0	1	0
48033551	C	G	MSH6	intronic	0	4	20	3	11	8	9	13	34
48033700	G	A	MSH6	non-synonymous SNV	0	1	0	2	0	0	0	2	0
178917005	A	G	PIK3CA	intronic	0	8	7	11	2	16	3	28	7
178921639	C	A	PIK3CA	intronic	0	12	16	11	9	15	14	24	25
178922274	C	A	PIK3CA	intronic	0	6	17	11	9	8	14	14	26
178927345	T	C	PIK3CA	intronic	0	5	1	5	0	5	0	9	1
178927410	A	G	PIK3CA	non-synonymous SNV	0	4	2	7	0	12	2	23	1
178947985	C	G	PIK3CA	intronic	1	0	0	0	0	0	0	1	0
178948196	A	G	PIK3CA	intronic	0	6	0	4	1	4	0	6	0
48575700	T	C	SMAD4	intronic	1	0	0	0	0	0	0	1	0
48584420	G	A	SMAD4	intronic	0	3	0	1	0	1	0	3	0
48584624	T	C	SMAD4	intronic	1	0	0	0	0	0	0	1	0
48591762	G	A	SMAD4	intronic	0	1	0	9	1	7	0	9	0
48592020	T	C	SMAD4	intronic	0	2	0	1	0	1	0	3	0
48602941	G	C	SMAD4	intronic	0	1	0	4	0	0	0	1	0
70117450	G	A	SOX9	intronic	0	3	0	1	0	0	1	3	1
70118935	C	T	SOX9	synonymous SNV	0	10	0	3	0	3	0	16	1
70120551	A	C	SOX9	intronic	0	13	1	8	0	11	3	19	0
114912081	G	A	TCF7L2	intronic	0	1	0	2	0	2	0	4	0
114920452	T	C	TCF7L2	intronic	1	0	0	0	0	2	0	0	0
30686414	A	G	TGFBR2	intronic	0	11	4	10	3	16	4	23	5
30713126	T	A	TGFBR2	intronic	0	6	0	10	0	12	0	13	1
30713619	C	T	TGFBR2	non-synonymous SNV	0	0	0	0	0	2	0	0	0
30713674	A	G	TGFBR2	synonymous SNV	0	6	0	3	0	3	0	10	0
30713842	C	T	TGFBR2	synonymous SNV	0	2	0	1	0	2	0	2	0
30729931	C	T	TGFBR2	synonymous SNV	0	2	0	0	0	0	0	2	0
7574003	G	A	TP53	stopgain	0	0	0	0	0	1	0	1	0
7576501	G	A	TP53	intronic	0	1	0	0	0	2	0	5	0
7577120	C	T	TP53	non-synonymous SNV	0	0	0	0	0	2	0	2	0
7578210	T	C	TP53	synonymous SNV	0	0	0	1	0	0	0	1	0
7578212	G	A	TP53	stopgain	0	0	0	0	0	0	0	4	0
7578406	C	T	TP53	non-synonymous SNV	0	1	0	0	0	0	0	3	0
7579311	C	A	TP53	intronic	0	0	0	0	0	0	0	1	0
7579472	G	C	TP53	non-synonymous SNV	0	21	0	14	0	22	0	34	0
7579801	G	C	TP53	intronic	0	23	0	12	1	20	0	36	0

#### *AMER1* variants

We found 461 variants in the discovery set of which 433 were novel. From these, 402 were non-synonymous, 25 were stopgains, and 6 were frameshift substitutions. All of these variants were in exon 2 of the *AMER1* gene. Four variants in the *AMER1* gene were validated on Illumina HiSeq of which 1 was novel. This variant at locus chrX: 63409685, a C to T change, had a frequency of 0.07 (2/30, both heterozygous) in normal tissue, 0.05 (1/21, heterozygous) in adenoma, 0.03 (1/33, heterozygous) in advanced adenoma and 0.02 (1/56, heterozygous) in CRC. (Table 2 and Figure 3B).

#### *ARID1A* variants

We found 742 variants in the discovery set, of which 695 were novel. From these, 653 were non-synonymous, 35 were stopgains, 6 were frameshift and 1 was a non-frameshift variant. Eight variants in the *ARID1A* gene were validated on the HiSeq platform of which 2 were novel (Table 2). Variant at locus chr2: 27106323, was synonymous, a C to T change, with a frequency of 0.03 (1/30, heterozygous) in normal and 0.07 (4/56, all heterozygous) in CRCs. Variant at locus chr2: 27105676 was a stopgain, a G to T change, with a frequency of 0.02 (1/56, heterozygous) in CRCs (Amino acid change from E to X). These variants were on exon 19 and 20 of the *ARID1A,* respectively. Three variants were mapped to N-terminus sequences before the ARID domain, 1 prior to the GR [the C-terminus of the protein can stimulate Glucocorticoid Receptor-dependent transcriptional activation] binding domain, and 4 in the GR binding domain (Table 2 and Figure 3C).

#### *FBXW7* variants

We found 262 variants in the discovery set, of which 230 were novel. From these, there were 210 non-synonymous and 20 stopgains. We validated five known variants. From these five, 2 were flanking intronic, and 3 were non-synonymous. Variant at locus Chr4: 153247289, exon 9, codon change of CGC to CAC had a frequency of 0.03 (1/33, heterozygous) in advanced adenoma and 0.04 (2/56, heterozygous) in CRCs, this variant was non-synonymous (Table 2). Non-synonymous variant at locus Chr4: 153247366, exon 9, a C to T change, had a frequency of 0.03 (1/33, heterozygous) in advanced adenoma. Variant at locus Chr4: 153245446, exon 10, codon change CAG to CAA, had a frequency of 0.03 (1/33, heterozygous) in advanced adenoma (Amino acid change from S to L), this variant was non-synonymous (Table 2). Four variants were mapped to the WD repeat domain while 1 was prior to the D domain (Figure 3D).

#### *TCF7L2* variants

We found 194 variants in the discovery set, of which 186 were novel. From these, 178 were non-synonymous, 5 were stopgains, 2 were frameshift substitutions, and 1 was stoploss. We validated two of these variants: 1 was novel flanking intronic at locus Chr10: 114920452, a T to C change, with a frequency of 0.06 (2/33, both heterozygous) in advanced adenoma. The other variant at locus Chr10: 114912081 was known, a G to A change, had a frequency of 0.03 (1/30, heterozygous) in normal tissue, 0.10 (2/21, both heterozygous) in adenoma, 0.06 (2/33, both heterozygous) in advanced adenoma, and 0.07 (4/56, all heterozygous) in CRCs (Table 2). One variant was mapped to the HMG box while 1 was to the activation domain (Figure 3E).

#### *TGFβR2* variants

We found 169 variants in the discovery set, of which 157 were novel. From these, 142 were non-synonymous and 15 were stopgains. We validated 6 known variants (Table 2). Three were synonymous at locus Chr3: 30713674 exon 4 with codon change of CTA to CTG with a frequency of 0.20 (6/30, all heterozygous) in normal, 0.14 (3/21, all heterozygous) in adenoma (No Amino acid change), 0.09 (3/33, all heterozygous) in advanced adenoma, and 0.18 (10/56, all heterozygous) in CRC; variant at locus Chr 3: 30713842 exon 4 with codon change C to T with a frequency of 0.07 (2/30, both heterozygous) in normal, 0.05 (1/21, heterozygous) in adenoma, 0.06 (2/33, both heterozygous) in advanced adenoma, and 0.04 (2/56, both heterozygous) in CRC; variant at locus Chr: 30729931 exon 6 with codon change of GTC to GTT with a frequency of 0.07 (2/30, both heterozygous) in normal, and 0.04 (2/56, both heterozygous) in CRC; and 1 was non-synonymous at locus chr: 30713619 exon 4 with codon change of ACG to ATG with a frequency of 0.10 (2/33, both heterozygous) in advanced adenoma (Table 2) (Amino acid change from T to M). The other 2 were flanking intronic. Four variants were mapped to the Cyt ProtKinase I, II and III domains, and 2 on the extracellular domain (Figure 3F).

#### *TP53* variants

We found 162 variants in the discovery set, of which 51 were novel. From these, 49 were non-synonymous, 1 was stopgain, and 1 was frameshift variant. We validated 9 variants that were all known. There were 3 non-synonymous variants at locus Chr17: 7579472 exon 3 with nucleotide change G to C and a frequency of 0.70 (21/30, all heterozygous) in normal, 0.67 (14/21, all heterozygous) in adenoma, 0.67 (22/33, all heterozygous) in advanced adenoma, and 0.61 (34/56, all heterozygous) in CRC (Amino acid change from P to R); locus Chr17: 7578406 exon 1 with nucleotide change C to T with a frequency of 0.03 (1/30, heterozygous) in normal, and 0.05 (3/56, all heterozygous) in CRC (Amino acid change from R to H); and locus Chr17: 7577120 exon 4 with nucleotide change C to T with a frequency of 0.04 (2/56, both heterozygous) in CRC (Amino acid change from R to H). There was one synonymous variant at locus Chr17: 7578210 exon 2 with codon change of CGA to CGG with a frequency 0.05 (1/21, all heterozygous) in adenoma, and 0.02 (1/56, all heterozygous) in CRC. There were two stopgain variants at locus Chr17: 7578212 exon 2 with codon change GCT to ACT with a frequency of 0.07 (4/56, all heterozygous) in CRC (Amino acid change from R to X); and locus Chr17: 7574003 exon 6 with nucleotide change G to A with a frequency 0.03 (1/33, all heterozygous) in advanced adenoma, and 0.02 (1/56, all heterozygous) in CRC (Amino acid change from R to X). The remaining 3 variants were intronic (Table 2). We mapped 1 variant to the transactivation domain, 1 in the proline rich domain, 5 in the DNA binding domain, and 2 in the oligomerization domain (Figure 3G).

#### *SMAD4* variants

We found 196 variants in the discovery set, of which 159 were novel. From these, 153 were non-synonymous, 4 were stopgains, and 2 were frameshift variants. We validated six intronic variants of which 2 were novel (Table 2). Variant at locus chr8: 48584624 with a T to C change had a frequency 0.02 (1/56, heterozygous) in CRCs. Variant at locus chr8: 48575700 with a T to C change had a frequency 0.02 (1/56, heterozygous) in CRCs. Of the 6 validated variants, three were found in the linker region and 3 in the MH2 region (Figure 3H).

### Oncogenes

#### *KRAS* variants

**Figure 4:**
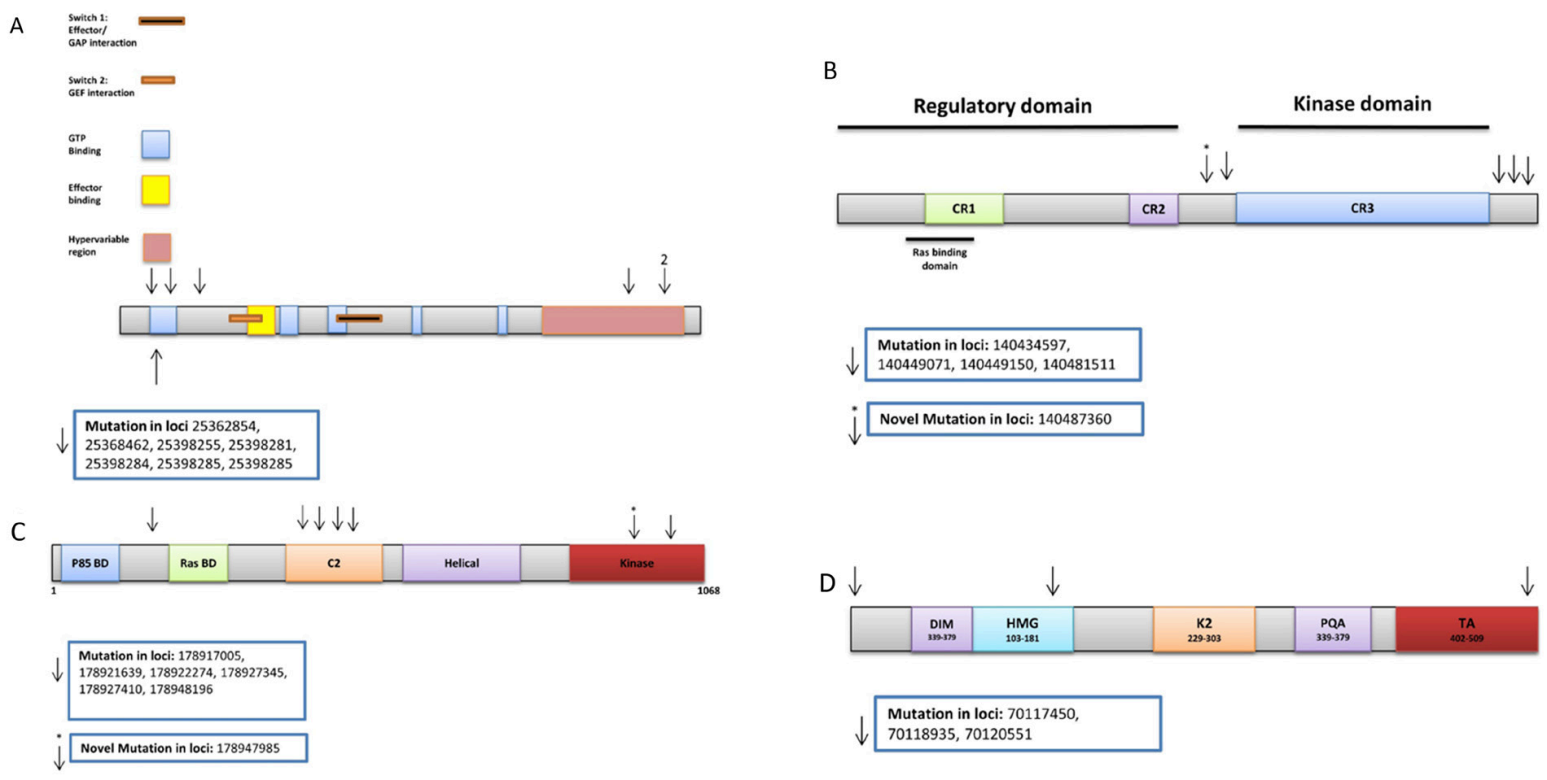
Oncogenes’ validated variants: **(A)** *KRAS,* **(B)** *BRAF,* **(C)** *PIK3CA,* **(D)** *SOX9*.

We found 54 variants in the discovery set, of which 44 were novel. From these, 41 were non-synonymous and 3 were stopgains. All variants were in the RAS domain. We validated seven known variants, 5 of which were non-synonymous, 1 synonymous, and 1 flanking intronic. The non-synonymous variants are as follows: locus 25398281 exon 2, a C to T change, with a frequency of 0.14 (3/21, all heterozygous) in adenoma, 0.15 (5/33, all heterozygous) in advanced adenoma, and 0.16 (9/56, all heterozygous) in CRC (Amino acid change from G to D); locus 25398284 exon 2, a C to T change, with a frequency of 0.14 (3/21, all heterozygous) in adenoma, 0.06 (2/33, both heterozygous) in advanced adenoma, and 0.21 (12/56, all heterozygous) in CRC (Amino acid change from G to D); locus 25398285 exon 2, a C to A change, with a frequency of 0.06 (2/33, both heterozygous) in advanced adenoma, and 0.02 (1/56, heterozygous) in CRC (Amino acid change from G to C); locus 25398285 exon 2, a C to T change, with a frequency of 0.09 (3/33, all heterozygous) in advanced adenoma, and 0.04 (2/56, both heterozygous) in CRC (Amino acid change from G to S); locus 25398255 exon 2 with nucleotide change G to T, and a frequency of 0.03 (1/33, heterozygous) in advanced adenoma (Table 2). Three variants were mapped in the GTP binding region, 1 C-terminal to the GTP binding region, (Amino acid change from Q to K) and 3 in the hypervariable region (Figure 4A).

#### *NRAS* variants

We found 57 variants in the discovery set, of which 50 were novel. From these, 44 were non-synonymous, 2 frameshift substitutions, and 4 stopgains. No NRAS variants were validated on the Illumina platform.

#### *BRAF* variants

We found 221 variants in the discovery set, of which 200 were novel. From these, 184 were non-synonymous and 16 were stopgain variants. We validated 5 variants of which 1 was novel. One variant was non-synonymous, 1 synonymous, and 3 flanking intronic. Non-synonymous novel variant Chr7: 140487360 exon 9 has a frequency of 0.02 (1/56, heterozygous) in CRC. Synonymous variant Chr7: 140449150 exon 16 has a frequency of 0.77 (23/30, 13 homozygous and 10 heterozygous) in normal, 0.76 (16/21, 5 homozygous and 11 heterozygous) in adenoma, 0.85 (28/33, 8 homozygous and 20 heterozygous) in advanced adenoma, and 0.86 (48/56, 18 homozygous and 30 heterozygous) in CRC (Table 2). The variants were mapped prior and after CR3 (Figure 4B).

#### *PIK3CA* variants

We found 251 variants in the discovery set, of which 214 were novel. From these, 191 are non-synonymous and 23 were stopgain variants. We validated seven variants of which 1 was novel (Table 2). One variant was non-synonymous while the other 6 were flanking intronic. Non-synonymous variant at locus Chr3: 178927410 exon 7, an A to G change, had a frequency of 0.27 (6/30, 2 homozygous and 4 heterozygous) in normal, (7/21, all heterozygous), 0.33 in adenoma, 0.42 (14/33, 2 homozygous and 12 heterozygous) in advanced adenoma and 0.41 (23/56, 1 homozygous and 22 heterozygous) in CRC. One variant was mapped prior to Ras BD region, 4 in the C2 domain, and 2 in the kinase domain (Table 2 and Figure 4C).

#### *SOX9* variants

We found 189 variants in the discovery set, of which 183 were novel. From these, 162 were non-synonymous, 13 were stopgains, and 8 were frameshift variants. We validated three known variants; 1 synonymous and 2 flanking intronic (Table 2). Synonymous variant at locus Chr17: 70118935 had a frequency of 0.33 (10/30, all heterozygous) in normal, 0.14 (3/21, all heterozygous) in adenoma, 0.09 (3/33, all heterozygous) in advanced adenoma and 0.32 (17/56, 1 homozygous and 16 heterozygous) in CRCs (Table 2). One variant was mapped prior to the DIM region, 1 in the HMG region, and 1 in the TA region (Figure 4D).

### MMR genes

*MSH3*: We found 315 *MSH3* variants in the discovery set, of which 298 were novel. We validated 14 known variants. Five of these variants were non-synonymous and altered exons 10, 21, and 23. Two were synonymous with changes in exon 4 and 18. Variant at locus 79966029 with a G to A change, with a frequency of 0.23 (7/30, 1 homozygous and 6 heterozygous) in normal, 0.52 (11/21, all heterozygous) in adenoma, 0.21 (7/33, 2 homozygous and 5 heterozygous) in advanced adenoma, and 0.29 (16/56, all heterozygous) in CRCs. Variant at locus 80083459 with a G to A change, with a frequency of 0.03 (1/30, heterozygous) in normal, 0.06 (2/33, both heterozygous) in advanced adenoma, and 0.02 (1/56, heterozygous) in CRCs. The rest were flanking intronic (Table 2). The variants were mapped to the MSH3-MSH2-MSH6 region with 3 variants prior to EXO1, 3 in EXO1, 1 in MutS_I, 3 in MutS_II, 1 in MutS_III, and 3 in MutS_V [5] (Figure 2A).

*MSH6*: We detected 434 variants in the discovery set, of which 396 were novel. The Illumina sequencing led to the validation of 12 variants. Two of these were novel. The A>G and A>C variants are flanking the region coding for the MutS-V domain and the MSH2 binding site, respectively. An A>C intronic variant (IVS8-45) at locus chr2:48033545 was observed in 1 CRC sample with a frequency of 0.02 (1/56, heterozygous). The A>G variant at locus chr2:48032908 was intronic (IVS7-50) and was observed in 1 sample with a frequency of 0.03 (1/30, heterozygous) in normal, 0.05 (1/21, heterozygous) in adenoma, and 0.04 (2/56, both heterozygous) in CRC (Table 2). One variant was mapped in PWWP, 2 prior to the MutS_I, 1 in MutS_III, and 8 in P-loop_NTPase [5] (Figure 2B).

### Summary of validated variants

In total, there were 12 validated variants in the 14 genes: *APC* (33/121 [27%]), *AMER1* (4/121) [3%])*, ARID1* (8/121 [7%]), *MSH3* (14/121 [12%]), *MSH6* (12/121 [10%]), *BRAF* (5/121 [4%]), *KRAS* (7/121 [6%]), *FBXW7* (5/121 [4%]), *PIK3CA* (7/121 [6%]), *SMAD4* (6/121 [5%]), *SOX9* (3/121 [2%]), *TCF7L2* (2/121 [2%]), *TGFBR2* (6/121 [5%]), *TP53* (9/121 [7%]). From these validated variants, 14/121 (12%) were novel variants in 8 genes *(AMER1, APC, ARID1A, BRAF, MSH6, PIK3CA, SMAD4,* and *TCF7L2*). Of the validated variants, 23% (28/121) were non-synonymous, 14% (17/121) were stopgains, 24% (29/121) were synonymous and 39% (47/121) were intronic variants.

## DISCUSSION

We performed a targeted exome sequencing in African Americans with colorectal lesions with the goal of detecting particular variant profiles that can partially explain the observed CRC disparity in this population through the identification of highly pathogenic and frequent variants and mutations. While most genes displayed a high number of variants (novel and known) on one platform (Ion Torrent), not all were validated on the second sequencing platform (HiSeq, Illumina). We previously reported such discrepancies on our recent study [33] thus mandating a necessary validation step that we performed on the HiSeq platform. We validated 121 variants in 14 mechanistically known driver genes including Tumor Suppressor Genes (TSGs): *APC* (33/121 [27%]), *AMER1* (4/121) [3%])*, ARID1* (8/121 [7%]), *FBXW7* (5/121 [4%]), *SMAD4* (6/121 [5%]), *TCF7L2* (2/121 [2%]), *TGFBR2* (6/121 [5%]), *TP53* (9/121 [7%]), oncogenes: *PIK3CA* (7/121 [6%]), *BRAF* (5/121 [4%]), *KRAS* (7/121 [6%]), *SOX9* (3/121 [2%]), and MMR genes, *MSH3* (14/121 [12%]), *MSH6* (12/121 [10%]). From these validated variants, 14/121 (12%) were novel variants in 8 genes *(AMER1, APC, ARID1A, BRAF, MSH6, PIK3CA, SMAD4,* and *TCF7L2*). Of the validated variants, 23% (28/121) were non-synonymous, 14% (17/121) were stopgains, 24% (29/121) were synonymous and 39% (47/121) were intronic variants. A sizable portion of the validated variants (39%) were intronic. While it is difficult to assess the potential effects of such variants on the protein product and function, many studies have shown that such variants lead to aberrant splicing with different levels of impact on the protein function in many pathological diseases [34].

Here, we report the profile of variants in African American colorectal lesions using targeted exome sequencing. Validated variants showed that the tumor suppressor genes *APC* and *ARID1* and the DNA MMR genes *MSH3* and *MSH6* are the genes with the highest numbers of validated variants. Oncogenes *KRAS and PIK3CA* were also primary variant targets that likely participated in increased proliferative potential of the mutated colonic epithelial cells in this population. Many of these recurrent and frequent variants were novel and not previously reported in any of the known databases.

The 8 TSGs (*AMER1, APC, ARID, FBXW7, TCF7L2, TGFBR2, SMAD4,* and *TP53)* in our gene panel accounted for 59% of the validated variants. If added to the 20% of validated DNA MMR genes’ variants, this will give a 73% rate of validated variants within TSGs. This finding is in line with the importance of TSGs in the initiation of the carcinogenic process and with the fact that such genes need double-hits for complete inactivation, unlike oncogenes. More than 2/3 TSGs validated variants in our study reflects perfectly the roles and rates at which such genes are targeted in the neoplastic transformation process.

*APC* is part of WNT pathway in CRC and was the top target gene with 27% (33/121) of the validated variants (4 novel variants, Table 3). Its role for downstream signaling with B-catenin, GSK and AXIN has been well established in previous studies [35]. Wnt/β-catenin pathway plays multiple and diverse roles in development by regulating gene expression via T-cell factor/Lymphoid enhancer-binding factor (Tcf/Lef) DNA binding factors [36]. Angus-Hill et al. showed that *Tcf4* (*Tcf7L2*) functions as a tumor suppressor gene in colon carcinogenesis [36].

**Table 3:** Distribution of validated variants in signaling pathways

Gene	Signaling Pathway	TSG vs. Oncogene	Function	Total # of Variants (n=121)	Total Variant FRQ	Novel Variant n, %
**AMER1**	Wnt/β-catenin	TSG	Proliferation, subcellular distribution of APC	4	3%	1, 1
**APC**	Wnt/β-catenin	TSG	Proliferation	33	27%	4, 3
**ARID1A**	Wnt/β-catenin	TSG	Proliferation	8	7%	2, 2
**BRAF**	RTK-RAS	Oncogene	Cell survival, translation, proliferation	5	4%	1, 1
**FBXW7**	Wnt/β-catenin	TSG	Proliferation	5	4%	No
**KRAS**	RTK-RAS	Oncogene	Cell survival, translation, proliferation	7	6%	No
**MSH3**	Mismatch repair	Suppression of Tumor	Mismatch repair	14	12%	No
**MSH6**	Mismatch repair	Suppression of Tumor	Mismatch repair	13	11%	2, 2
MMR (MSH3^*^, MSH6, MSH2, PMS2, MLH1	Mismatch repair	Suppression of Tumor	Mismatch repair	27	23%	2, 2
**PIK3CA**	PI3K-Akt	Oncogene	Cell survival, translation, proliferation	7	6%	1, 1
**SMAD4**	Wnt/β-catenin	Oncogene	Proliferation	6	5%	2, 2
**SOX9**	Wnt/β-catenin	Oncogene	Proliferation, Self-Renewal of Oncogene Targeted Cells	3	2%	No
**TCF7L2**	Wnt/β-catenin	Both (TSG/ Oncogene)	Proliferation	2	2%	1, 1
**βR2**	TGF-β (growth factor)	TSG	Proliferation	6	5%	No
**TP53**	P53	TSG	Proliferation, Cell survival	9	7%	No

Exome Sequencing has revealed *AMER1* as a frequent target in CRC [37]. In our study, we report one novel variant with a frequency of 0.07 in normal, 0.05 in adenoma, 0.03 in advanced adenoma and 0.02 in CRC samples. Overexpression of *AMER1* increases the expression of APC and causes subcellular re-localization of APC from the microtubule ends to the plasma membrane of epithelial cells [37]. As such, variants within this gene may downregulate the APC gene and downstream signaling pathways. *SMAD4* is a well-established tumor suppressor gene that displayed a high number of novel variants (n=112), however none were validated on the Illumina platform. Only 4 known variants were validated and they were all mapped to the linker region (Figure 3H).

*ARID1A* has been identified as a novel tumor suppressor gene in ovarian cancer and subsequently in various other tumor types. *ARID1A* encodes a protein that belongs to the ARID domain containing family, which consists of 15 genes encoding proteins involved in transcriptional regulation, proliferation and chromatin remodeling [38]. There were 7% (8/121) validated variants highlighting a potential bigger role of this TSG in CRC in African Americans (Table 2, Figure 3C).

TGFBR2 signaling is involved in cell-cell communication, cell adhesion and cell migration. The role of this pathway in the glycosylation pattern of cell surface proteins is largely unexplored. Experimental evidence suggested a possible link between mutated MSI target genes and the glycosylation pattern at the cell surface [39]. We detected 5% (6/121) of the variants in TGFBR2 gene (Table 3), 1 non-synonymous variant at locus Chr: 30713619 exon 4 with codon change of ACG to ATG that mapped to the Cyt ProtKinase I, II and III domain (Figure 3F). This variant could have an effect on tumor invasive phenotype.

*FBXW7* has been identified as a transcriptional target of TP53 and lower expression levels of *FBXW7* in correlation with *TP53*-variants have been reported [40]. *FBXW7* loss leads to induction of p53-phosphorylation at Serine-15, p53 stabilizes in the nucleus to act as a transcriptional activator for tumor suppression, implicating phospho-p53 (Ser15) as a marker of *FBXW7*-associated carcinogenesis [27]. We found intronic and non-synonymous variants both in advanced adenoma and in CRCs (Table 2). All four variants were mapped to the WD repeat domain of the protein (Figure 3D). The mutations in this gene may contribute to the decreased efficacy of therapy in FBXW7-mutated CRC [27].

*TP53* is frequently inactivated by variant or deletion in most human tumors [41]. A tremendous effort has been made to restore TP53 activity in cancer therapies. However, no effective p53-based therapy has been successfully translated into clinical cancer treatment owing to the complexity of p53 signaling [41]. For the *TP53* gene, there were 7% (9/121) validated that were mapped (Figure 3G). The potential deleterious effect of these variants needs experimental validation.

The oncogenes within the sequenced gene panel had a total of 22 out of 121 validated variants *(PIK3CA (7/121 [6%]), BRAF (5/121 [4%]), KRAS (7/121 [6%]), SOX9 (3/121 [2%])).* We previously reported on variants targeting *BRAF* and *KRAS* in African Americans with colorectal cancer, however the present data seems to give weight to other oncogenes such as *PIK3CA* as major targets of variants.

Establishing *KRAS* variants’ status in each patient is indeed important to determine the appropriate therapy, patients with wild-type *KRAS* could receive monoclonal antibodies against EGFR [42] while *KRAS* mutated patients have been associated with no-response to targeted therapies and poor prognosis in different studies [43]. In our study, we found 1 non-synonymous known variant with a 0.03 frequency in patients with advanced adenoma (Table 2). There were no detected variants in the notable hotspots of *KRAS* (codons 12/13/61/144) [44].

Variants in the *BRAF* oncogene is a key step in malignant transformation within a subset of CRCs, generally MSI-H and with the CpG methylator phenotype [16]. It is unclear to what extent the lack of response in *KRAS* wild-type CRCs is due to *BRAF* variants, but data suggest that mutated *BRAF* confers resistance to anti-EGFR therapy [45, 46]. According to Palmirotta et al., a *KRAS* variant (CAG>TAG) determining a premature stop signal at codon 22 (Gln22Stop) has been previously found in a patient with metastatic colorectal cancer [47]. Whether or not the validated stopgain variants in *KRAS* associate with an activating effect remains to be explored and investigated.

The prevalence of *PIK3CA* variants increases continuously from rectal to cecum cancers, supporting the ‘colorectal continuum’ paradigm, and an important interplay of gut microbiota and host immune/inflammatory reaction [48]. *PIK3CA* variants also contribute to significantly decreased survival for patients with wild-type *BRAF* tumors [49]. Numerous studies have shown that variants are concentrated in 2 hotspots of the *PIK3CA* gene: the helical domain in codons 542 and 545 of exon 9 and the kinase domain in codon 1047 of exon 20 [49]. In our study, *PIK3CA* variants were 6% of the validated variants (Table 2). The novel variant was intronic with a CRC frequency of 0.02 (Table 2). This variant was mapped to N-terminus region before the Kinase region of the PIK3CA. The known variant was also intronic with a variants’ frequency of 0.2 in normal, 0.29 in adenoma, 0.12 in advanced adenoma, and 0.11 in CRCs (Table 2). Recently Kim et al, showed that *PIK3CB* rare point variant is associated with tumorigenesis [50]. This somatic variant event in the cancer, supports our results that low frequency of the variants may have tumorigenic function that needs verification with *in vivo* and *in vitro* analysis.

In the intestinal epithelium, *SOX9* is expressed in the stem/progenitor cells, as well as in the nuclei of terminally differentiated Paneth cells of the small intestinal crypts and tuft cells in the villi. It plays a crucial role in Paneth cell differentiation [19, 51]. From our panel, 2% (3/124) of validated variants were in this gene. This corresponds to an intronic variant with a frequency of 0.1 in normal, 0.05 in adenoma, 0.06 in advanced adenoma and 0.09 in CRC samples (Table 2). This variant was mapped to the DIM region of SOX9 (Figure 4D).

Among MMR genes, 26 of the validated variants were in *MSH3* and *MSH6* genes accounting for 22% of the validated variants [5]. This finding highlights the important role that these genes play in the setting of genome-wide instability aside from the already well described roles of *MLH1* and *MSH2* genes in this process. Indeed, variants within these genes, and specifically within *MSH3*, have been linked to the EMAST phenotype characterized by instability within tetranucleotide repeats. Patients with such phenotype have a poor prognosis. This might help partly understand colorectal cancer disparity in African Americans. Indeed, EMAST is reported to be highly prevalent (50 to 60%) in this population when compared to others [11].

The lack of matching normal for many of the analyzed the samples, and the absence of *MSH2*, *POLE*, *MLH1* and *SMAD2* on our targeted exome panel constitute limitations to our study. However, our study is a good example for other more comprehensive studies that will use larger sample size and more inclusive gene panels particularly for African American patients which is under-represented.

In this study, we examined and validated variants in 15 driver cancer genes in African Americans with colorectal neoplastic lesions. Many of these driver genes are involved and different pathways are affected by these variants which are all part of the carcinogenic process. Among oncogenes and TSGs, *PIK3CA* and *APC* genes were the most frequently altered genes, respectively. DNA MMR genes *MSH3* and *MSH6* also displayed a high level of variants that probably affect overall genetic stability. The distinct variant profiles with novel variants can help to predict, diagnose and establish new targeted therapeutic modalities for optimal CRC patients’ care.

## MATERIALS AND METHODS

### Discovery set

A total of 140 colorectal samples including 30 normals, 21 adenomas, 33 advanced adenomas (> 1 cm and/or with villous histology), and 56 cancers were used to establish variant profiles by targeted exome sequencing, using a PGM sequencing platform (Figure 1). These samples were collected from 123 patients at different stages of the disease. The Howard University Institutional board approved (06-MED-39) the collection of archival unidentified human samples.

### Validation set

A total of 36 samples from 26 patients including 12 normals, 5 adenomas, 4 advanced adenomas and 15 cancers, were used to validate the Ion Torrent detected variants on a second sequencing platform (HiSeq, Illumina). The validation set is a subset from the discovery set samples (Figure 1).

### Proton (Ion Torrent) NGS

A targeted, multiplex PCR panel was designed using the custom Ion AmpliSeq Designer v1.2 (ThermoFisher Scientific, Grand Island, NY). The panel amplified 56.9 kb and included the coding regions of 20 genes, with an average coverage of 96.9% of the protein coding regions and splice junctions (+5 intronic bases). In this study, we only report data from the 15 genes that are common to the Illumina gene panel that were sequenced in the validation set. The panel was designed to amplify PCR products with an average amplicon size of 150 base pairs (bp). Sample DNA (20 ng/primer pool) was amplified using the PCR panel, and libraries were prepared using the AmpliSeq Library Preparation kit following the manufacturer’s protocol (Thermo-Fisher Scientific, Grand Island, NY). Individual samples were barcoded, pooled, and sequenced on a Proton Sequencer using the Ion PI Template OT2 200v3 and Ion PI Sequencing 200v2 kits per the manufacturer’s instructions. Raw sequencing reads were filtered for high quality reads and the adaptors were removed using the Ion Torrent Suite 4.0.4, then reads were aligned to the hg19 reference sequence by TMAP (https://github.com/iontorrent/TS/tree/master/Analysis/TMAP) using default parameters. Resulting BAM files were processed through an in-house quality control (QC) filter and coverage analysis pipeline. BAM files were aligned using the GATK LeftAlignIndels module. Amplicon primers were trimmed from aligned reads using the Torrent Suite. Variant calls were made by the Torrent Variant Caller 4.0 (www.edgebio.com/variant-calling-ion-torrent-data).

### HiSeq (Illumina) NGS

DNA quantification and quality assessment for the validation set, NGS using a HiSeq platform (Illumina, San Diego, CA), SNV and indel detection, and assessment of copy number alterations were performed as previously described [5, 6].

### Bioinformatics and comparison of African Americans' variants to available databases

We used R software (version 2.15.2, http://www.r-project.org) to compare the variants in the normal and tumor samples with publicly available databases. Variants were annotated using ANNOVAR (56) and the 1000Genomes database, which represents a nominally noncancerous population. TCGA and GENIE databases were used to compute the frequency of mutations in another colorectal cancer database (TCGA) and a nominally non-cancerous population (GENIE). All samples displayed more or less an equal number of SNVs in their tumors compared with their matched normal samples. We would like to make it clear that variants identified using both platforms allowed us to identify weaknesses in bioinformatics associated with each platform that were addressed by rigorous manual review that improved variant detection on each individual platform. Subsequent to incorporating the more rigorous review, we identified additional variants with high confidence using a single platform.
